# Leveraging Digital Technology to Overcome Barriers in the Prosthetic and Orthotic Industry: Evaluation of its Applicability and Use During the COVID-19 Pandemic

**DOI:** 10.2196/23827

**Published:** 2020-11-05

**Authors:** Trevor Binedell, Karupppasamy Subburaj, Yoko Wong, Lucienne T M Blessing

**Affiliations:** 1 SUTD-MIT International Design Centre Singapore University of Technology and Design Singapore Singapore; 2 Engineering Product Development Pillar Singapore University of Technology and Design Singapore Singapore; 3 Footcare and Limb Design Centre Tan Tock Seng Hospital Singapore Singapore; 4 Consortium for Clinical Research and Innovation Singapore Singapore Clinical Research Institutes Singapore Singapore

**Keywords:** rehabilitation, telehealth, telemedicine, 3D printing, additive manufacturing, prosthetics, orthotics, assistive technologies, amputee, stroke, virtual, COVID-19

## Abstract

**Background:**

The prosthetic and orthotic industry typically provides an artisan “hands-on” approach to the assessment and fitting of orthopedic devices. Despite growing interest in digital technology for prosthetic and orthotic service provision, little is known of the quantum of use and the extent to which the current pandemic has accelerated the adoption.

**Objective:**

This study’s aim is to assess the use of digital technology in prosthetics and orthotics, and whether its use can help overcome challenges posed by the current COVID-19 pandemic.

**Methods:**

A web-based survey of working prosthetists, orthotists, and lower limb patients was conducted between June and July 2020 and divided into three sections: lower limb amputees, prosthetist and orthotist (P&O) currently using digital technologies in their practice, and P&O not using any digital technology. Input was sought from industry and academia experts for the development of the survey. Descriptive analyses were performed for both qualitative (open-ended questions) and quantitative data.

**Results:**

In total, 113 individuals responded to the web-based survey. There were 83 surveys included in the analysis (patients: n=13, 15%; prosthetists and orthotists: n=70, 85%). There were 30 surveys excluded because less than 10% of the questions were answered. Out of 70 P&Os, 31 (44%) used digital technologies. Three dimensional scanning and digital imaging were the leading technologies being used (27/31, 88%), primarily for footwear (18/31, 58%), ankle-foot orthoses, and transtibial and transfemoral sockets (14/31, 45%). Digital technology enables safer care during COVID-19 with 24 out of 31 (77%) respondents stating it improves patient outcomes. Singapore was significantly less certain that the industry's future is digital (*P*=.04). The use of virtual care was reported by the P&O to be beneficial for consultations, education, patient monitoring, or triaging purposes. However, the technology could not overcome inherent barriers such as the lack of details normally obtained during a physical assessment.

**Conclusions:**

Digital technology is transforming health care. The current pandemic highlights its usefulness in providing safer care, but digital technology must be implemented thoughtfully and designed to address issues that are barriers to current adoption. Technology advancements using virtual platforms, digitalization methods, and improved connectivity will continue to change the future of health care delivery. The prosthetic and orthotic industry should keep an open mind and move toward creating the required infrastructure to support this digital transformation, even if the world returns to pre–COVID-19 days.

## Introduction

### Background

In prosthetic and orthotic facilities, there is a need for a combination of care and technical expertise. Prosthetic and orthotic services are generally delivered face-to-face with a high amount of physical contact. As a result, the pandemic provides unique challenges that can be difficult to overcome. Currently, the prosthetic and orthotic industry designs devices to restore, replace, correct, protect, or immobilize a body part through handcrafted artisan approaches. These devices are highly patient-specific and are a result from the specialized skills and experience of the individual prosthetist and orthotist (P&O) [1]. The provision of these prostheses and orthoses is time-consuming and wasteful, and not completely customized [2], with production costs a burden [3]. They require ongoing maintenance and monitoring, and repeated visits to a clinic to ensure optimal fit and function throughout their use.

The introduction of digital technologies aims to improve these inefficiencies. Digital technology in this paper refers to 3D scanners, tablets, computers, computer cloud-based software programs, and computer-aided design and manufacturing (CADCAM). Virtual care refers to the use of telehealth, telerehabilitation, virtual assessments, and fittings. Digital technology and virtual care have successfully provided assistive devices assessment [4,5], therapy services [6], and diagnostic evaluations [7]. They have also eliminated distance obstacles from health care [8]. Digital technology offers possible solutions to patient care during the current pandemic, as health care systems try to limit the spread of COVID-19 by minimizing patient contact and improving hygiene practices [9].

### Digitalization of the Prosthetic and Orthotic Design and Manufacturing Process

To reduce the risk of COVID-19 spread, emerging protocols are advising for less physician-patient contact, shortening the contact time, and keeping a safe distance. It is recommended that unnecessary casting of patients be avoided and that alternative methods be used [10]. Three dimensional scanning is one such method and provides high accuracy [11], reduces product waste, and improves quality [12,13]. It has a high capability to capture 3D measurement without physical contact [14,15] and minimize the need for messy plaster of Paris casting. Digital libraries of files are created, manipulated, and personalized to fit a patient’s unique needs with greater precision and ease [16,17]. These files can be either outsourced for central fabrication via CADCAM technologies or printed using additive manufacturing systems. Three dimensional scanning and printing are currently used in applications across a spectrum of devices that include ankle-foot orthoses [18,19], helmets [20,21], and prostheses [22-24].

The use of CADCAM in the prosthetic and orthotic applications has been rapidly developing as a technology since the mid-1980s [25]. Although considered expensive to use due to the high infrastructure and equipment costs, the technology has shown great potential [26] but requires users with significant computer-aided design (CAD) experience [27]. The technology’s benefits during COVID-19 include reducing the contact time spent with the patient or coworkers and for use in satellite clinics where central fabrication facilities can quickly produce the prostheses or orthoses and have them shipped to the provider [26]. It also delivers shorter waiting times, design consistency, repeatability, quantifiable modification, and modern manufacturing [28,29].

### Digitalizing Assessment and Care

Despite its benefits to improve outcomes and use the contactless process of scanning to reduce cross-contamination [14,15], the use of digital technology is not without challenges to routine clinical care. There are often high capital costs in equipment and training, and concerns over the return of investment. Researchers still debate the ideal way to “digitize” the residual limb, whether it is better to cast and scan the negative mold, whether medical imaging (computed tomography [CT], magnetic resonance imaging [MRI], or x-ray) is more suitable [30,31], whether scanning should be done while weight-bearing [25], or not. An “expert” P&O has little to gain in the short run by adopting computerized methods [26]. A significant amount of retraining is required, and current virtual technology has not overcome typical physical characteristics of an assessment such as palpation.

Prosthetic and orthotic patient treatment during the current pandemic with digital technology has opened up the possibility for virtual measurements, fitting, and home-based rehabilitation [32-36]. Bringing care to the patient rather than the patient to care provides a safer environment for patients. The use of a mobile phone that includes inertial sensors and gyroscopes has the potential to overcome the physical assessment and contact usually associated with a consultation. The apps developed for mobile phones have shown use in measuring steps, balance, range of motion (ROM), education, and the provision of exercise programs [32,34,37] but are rarely used for assessment of patients requiring prostheses or orthoses [38,39].

Virtual care offers a unique capacity for remote screening, triage, and treatment. It could be a powerful tool for reducing transmission of contagious diseases such as COVID-19 to and among health care workers and patients who are not infected [40]. With patients using the internet to access health care increasing each year, the quality of any service provided by this means should be evidence-based and necessary [41]. Any assessment administered online needs to be followed by automated reports with scans or images, objective and subjective assessment [42,43], patient expectations, prescription, expected outcomes, and timelines. Virtual assessment can overcome many of the pitfalls of physical assessment while greatly expanding the potential pool of patients who may be unable or unwilling to attend a physical clinic. Due to the current pandemic crisis, the British Association of Prosthetics and Orthotics recommends the use of virtual care for triage, advice, assessment, reviewing ongoing care, the provision of off-the-shelf orthoses, and the review of all patients undertaking virtual assessments once normal working conditions resume [44].

Several barriers exist in virtual care implementation, including the lack of reimbursement [45,46], patient privacy and confidentiality, medicolegal concerns, practical workflow concerns, and physicians’ fears of being overwhelmed by online messages [47]. Furthermore, virtual assessments lack the vital elements of palpation, dynamic testing, and real-time feedback for the P&O. Some patients may also find virtual assessments impersonal and may feel more comfortable seeing someone in person to get the care they need. There remains the ongoing issue of internet connectivity in some regions, the high cost of hardware and software, and the patient’s ability to use information technology (IT). The quality of service for livestreaming audio and video applications must be improved to provide sustained bandwidth and low latency [8].

### Prosthetic and Orthotic Care Under COVID-19

Novel technologies like telemedicine may be useful in maintaining social distancing, monitoring a patient’s condition, or detecting infectious diseases, protecting not only patients but also health care providers [9]. This kind of virtual care can also address several aspects of assessment and care that do not require the time and effort necessary to travel to the P&O or allow care when such travel is not possible or puts patients at risk, such as during a pandemic. The current COVID-19 situation necessitates that we use available resources to optimize patient quality and outcome of the virtual visit [27]. The result of this pandemic has propelled virtual care adoption and transformed health care delivery [40].

The delivery care model will need to change as a result of COVID-19. There may be a “new normal” that is different from traditional practice, including the increased use of digital technologies. Digital technologies can potentially lead to different and more efficient designs, provide greater access to care, and limit physical contact. However, digital technology must be implemented thoughtfully and designed to address issues that are barriers to current adoption.

This paper presents the results of a study aimed at assessing the applicability and barriers of digital technology use in prosthetics and orthotics, and whether this technology can help overcome challenges posed by the current COVID-19 pandemic on the industry.

## Methods

An online survey was designed and used to survey P&Os currently practicing and lower limb amputees using a prosthesis on their use and attitudes toward digital technology. This study was approved by the Institutional Review Board (IRB) at Singapore University of Technology and Design. Interested participants agreed to a preceding statement of consent, and a participant information sheet link was provided describing the survey, including length of the survey, purpose of the study, investigators, and how data would be collected and stored. The survey was hosted and all data stored on a secure server. Participants were asked for their email only if they agreed to a follow-up interview. This information was stored separately from the responses to maintain confidentiality. Participants were able to review and change their answers before submission. The survey was developed by the authors in conjunction with ProsFit Technologies, Bulgaria and tested with five Singaporean P&Os. This data was not included in the final analysis but was analyzed to adjust the survey for any errors.

The survey was open to participants who met the inclusion criteria. The survey was administered between June and July 2020 via the SurveyMonkey platform and was voluntary to complete. Participants were recruited via IRB-approved social media platforms like LinkedIn, WhatsApp, and social chat groups.

The 68 items of the qualitative and quantitative survey were divided into three sections, with adaptive questioning routing the participant to questions based on previous responses. The first section of the survey gathered lower limb amputees’ (LLA) experiences and preferences. This included questions relating to prosthetic use, barriers to care, and opinions on the use of virtual assessments and home fittings. Section two was designed for the P&O who did not use digital technology (P&O-nonDT) in their facility. Questions included the number of patients seen per day, attitudes toward digital technology, and its importance to the future of the profession. Section three was for P&O who are currently using digital technology (P&O-DT) in their facilities. Additional questions about the use and limitations of technology were included in this section.

All three sections included demographic questions and questions on the use of virtual assessments or fittings. A variety of formats were used: multiple choice with single or multiple answers, ranking of answer options, 5-point Likert-scale questions, and open-ended questions. Where options were provided, the option “Other” was included to allow respondents to enter a different answer.

Follow-up interviews were conducted on selected patients and P&O respondents. Interviews were unstructured and conducted face-to-face or via phone and email.

Survey responses were analyzed with Stata/SE software (StataCorp LLC). Time stamps were collected at the start and end of the survey. All tests were carried out using a 5% level of significance. Answer options were presented as counts (%), mean (SD), or median (IQR) as appropriate. The Pearson chi-square test was used to assess difference between frequencies as observed and expected for certain answers.

## Results

### Participants

We received 113 survey responses, of which 83 were eligible for inclusion (n=13 LLA; n=70 P&Os). Surveys were excluded if less than 10% of the questionnaire was answered. On average, the survey took 13 minutes for the P&O to answer and 15 minutes for the LLA to complete.

Table 1 shows the demographics of the respondents. Singapore was well represented; although only 18.6% of the respondents (n=13), this constitutes 68% of all P&O in Singapore. LLA were from Singapore (n=12) and India (n=1). Follow-up interviews were conducted with LLA from Singapore (n=3) and with P&O who were using at least one form of digital technology (P&O-DT) from Singapore (n=3), Thailand (n=2), Malaysia (n=1), and Cambodia (n=1).

**Table 1:** Demographics of the respondents.

Demographics				Prosthetists/orthotists (n=70), n (%)		Lower limb amputee (n=13), n (%)
**Age range (years)**						
	18-24			5 (7.1)		1 (7.7)
	25-34			33 (47.1)		2 (15.4)
	35-44			22 (31.4)		3 (23.1)
	45-54			8 (11.4)		4 (30.8)
	55-64			2 (2.9)		3 (23.1)
**Gender**						
	Male			41 (58.6)		13 (100)
	Female			29 (41.4)		0 (0)
**Country**						
	**Southeast Asia and Asia**			56 (80)		13 (100)
		Singapore	13 (18.6)		12 (92.3)	
		Myanmar	8 (11.4)		0 (0)	
		Thailand	8 (11.4)		0 (0)	
		Malaysia	7 (10)		0 (0)	
		Cambodia	6 (8.6)		0 (0)	
		Indonesia	4 (5.7)		0 (0)	
		Sri Lanka	4 (5.7)		0 (0)	
		India	3 (4.3)		1 (7.7)	
		Hong Kong	1 (1.4)		0 (0)	
		Philippines	1 (1.4)		0 (0)	
		Japan	1 (1.4)		0 (0)	
	**Middle East**			2 (2.9)		0 (0)
		Yemen	1 (1.4)		0 (0)	
		Saudi Arabia	1 (1.4)		0 (0)	
	**Europe**			8 (11.4)		0 (0)
		Bulgaria	2 (2.9)		0 (0)	
		UK	2 (2.9)		0 (0)	
		Germany	1 (1.4)		0 (0)	
		Ireland	1 (1.4)		0 (0)	
		Scotland	1 (1.4)		0 (0)	
		France	1 (1.4)		0 (0)	
	**Other**			4 (5.7)		0 (0)
		Australia	4 (5.7)		0 (0)	

Table 2 shows the characteristics of the LLA respondents. LLA were primarily of K3 and K4 activity levels in the US Medicare Functional Classification levels (12/13, 92%) and had their amputation due to trauma (8/13, 62%). They reported a long duration of daily use (mean 8.69, SD 5.12 hours) and a mean socket comfort score of 6.97 (SD 1.15). Out of 13 respondents, 11 (85%) LLA had their prostheses measured using plaster, and only 2 patients used only measurements. Zero LLA used scanning to make their prosthesis. LLA’s mobility was mostly impacted by pain, followed by the ease of wearing their prosthesis, their ability to access care, and the temperature.

**Table 2:** Characteristics of lower limb amputees.

Characteristics		Lower limb amputee (n=13)
K2: community ambulator, n (%)		1 (8)
K3: unlimited community ambulator, n (%)		7 (54)
K4: unlimited and recreational sports, n (%)		5 (38)
Nontrauma (cancer, diabetes, vascular disease), n (%)		5 (38)
Trauma, n (%)		8 (62)
**Hours of using prosthesis each day**		
	Range	0-18
	Mean (SD)	8.69 (5.22)
	Median (IQR)	8 (6.3)
**Level of comfort with a prosthesis (0=least comfortable, 10=most comfortable)**		
	Range	4-9.4
	Mean (SD)	6.97 (1.15)
	Median (IQR)	7.3 (1.5)
**Methods of casting, n (%)**		
	Plaster wrap	11 (84.62)
	Scanning	0 (0)
	Measurement alone	2 (15.38)
**Ranking of factors that most impact mobility, mean (SD)**		
	Pain	2.46 (1.89)
	Easy to wear	2.92 (1.85)
	Access to care	4.54 (1.51)
	Breathability/temperature	4.54 (1.90)
	Durability	4.69 (1.93)
	Stability	4.85 (2.91)
	Weight	4.92 (1.71)
	Appearance	7.08 (1.66)

Tables 3 and 4 shows the characteristics of the P&O respondents. The P&O had a mean of 9.33 (SD 7.37) working years. The mean number of patients seen per day was 5.81 (SD 4.28). Almost half of the P&O used digital technology (31/70, 44%). Singapore had more (11/13, 85%) P&Os use digital technology compared to Myanmar (0/8, 0%).

**Table 3:** Characteristics of prosthetist and orthotist respondents.

Characteristics		Prosthetist and orthotist (n=70)
**Years of working**		
	Range	1-32
	Mean (SD)	9.33 (7.37)
	Median (IQR)	7 (10.0)
**Number of patients seen per day**		
	Range	0-20
	Mean (SD)	5.81 (4.28)
	Median (IQR)	4 (6.0)
**Use of digital technology as part of work, n (%)**		
	Yes	31 (44.29)
	No	39 (55.71)
**Years using technology (n=31)**		
	Range	0.5-24
	Median	2

**Table 4:** Country of prosthetist and orthotist respondents.

Country			P&O-DT^a^ (n=31), n	P&O-nonDT^b^ (n=39), n
**Southeast Asia and Asia**			24	34
	Singapore	11		2
	Myanmar	0		8
	Thailand	4		4
	Malaysia	1		6
	Cambodia	1		5
	Indonesia	2		2
	Sri Lanka	1		3
	India	0		3
	Hong Kong	1		0
	Philippines	0		1
	Japan	1		0
**Middle East**			1	1
	Yemen	0		1
	Saudi Arabia	1		0
**Europe**			5	3
	Bulgaria	2		0
	UK	1		1
	Germany	0		1
	Ireland	1		0
	Scotland	1		0
	France	0		1
**Other**			3	1
	Australia	3		1

^a^P&O-DT: prosthetists and orthotists who are currently using digital technology.

^b^P&O-nonDT: prosthetists and orthotists who did not use digital technology.

### Use and Types of Technologies

The number of years the P&O-DT had been using digital technology varies greatly, from 0.5 to 24 years, with a median of 2 years. Many of the P&O had CADCAM facilities where they worked (23/31, 74%). The iPad with a structure scanner was the preferred method for digital capture (12/31, 39%) with a mix of other scanners used, including Artec Eva Lite, Omega, and Rodin 4D. Geometrical modification of the scans were performed using various programs, which can be grouped into P&O-specific software (24/31, 77%) and engineering software such as Rhinoceros or Solidworks (6/31, 19%). One P&O respondent was unsure of the program they used (1/31, 4%).

**Figure 1:**
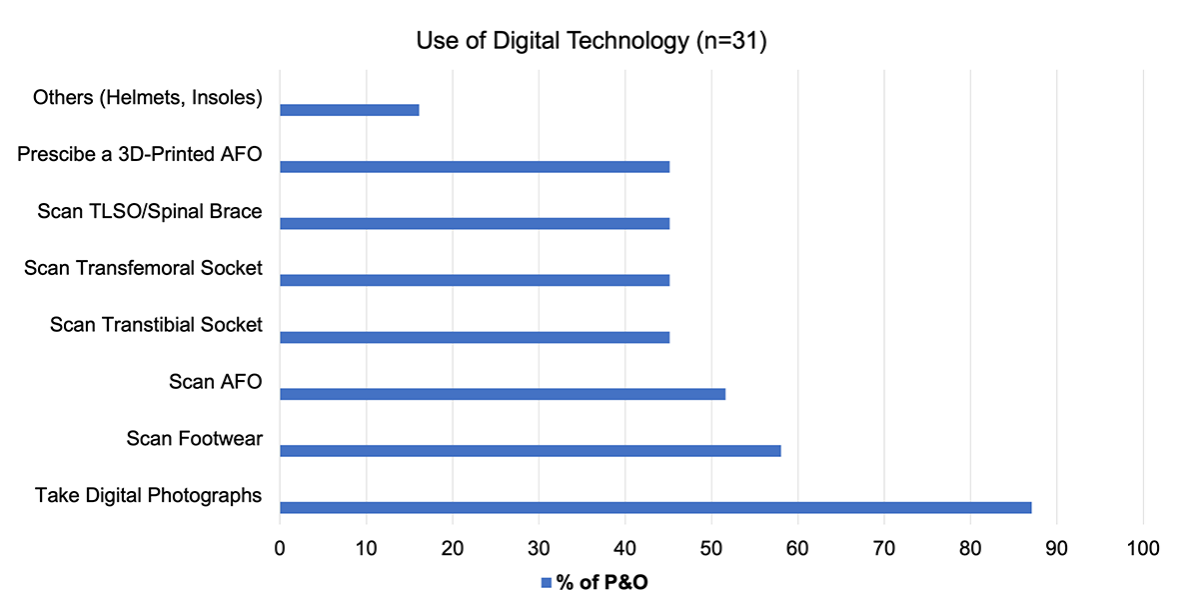
The applications of digital technology used in clinical practice. AFO: ankle-foot orthosis; P&O: prosthetic and orthotic; TLSO: thoracic-lumbar-sacral orthosis.

Figure 1 shows the application areas of the technology. Predominantly, the technology seems to show that taking digital photos to monitor care and to inform the design (27/31, 87%) is the most common use, followed by scanning for custom footwear (18/31, 58%). Approximately half of the subjects would scan for an ankle-foot orthosis (AFO), spinal braces, or transtibial or transfemoral sockets.

Five-point Likert-scale questions showed that the attitudes toward digital technology among P&O using technology were generally positive (see Table 5). Out of 31 respondents, 24 (77%) agreed or strongly agreed that it improves patient outcomes. The majority of participants agreed that they have the necessary skills to incorporate digital technologies (25/31, 81%) and acknowledged a strong need to continue using the technology to maintain efficacy and improve skills (30/31, 97%), and approximately two-thirds (20/31, 65%) were conscious that patients prefer them to use digital technology for their care. However, just over half (17/31, 55%) agreed that 3D printed devices were cost-effective, and 22 out of 31 (71%) felt that digitally produced prosthetic and orthotic devices did not fit better than traditionally made ones.

**Table 5:** Attitudes of prosthetists and orthotists who use digital technologies at work.

Attitudes		Total (n=31), n (%)	Singapore (n=11), n (%)	Non-Singapore (n=20), n (%)	*P* value	
**Digital technology improves patient outcomes**						.13
	Strongly agree	9 (29)	2 (18.2)	7 (35)		
	Agree	15 (48.4)	8 (72.7)	7 (35)		
	Disagree	7 (22.6)	1 (9.1)	6 (30)		
	Strongly disagree	0 (0)	0 (0)	0 (0)		
**Patients prefer me to use digital technology when making their devices**						.12
	Strongly agree	4 (12.9)	3 (27.3)	1 (5)		
	Agree	16 (51.6)	6 (54.6)	10 (50)		
	Disagree	11 (35.5)	2 (18.2)	9 (45)		
	Strongly disagree	0 (0)	0 (0)	0 (0)		
**It is important to practice with the hardware/software to be more efficient and effective**						.28
	Strongly agree	21 (67.7)	8 (72.7)	13 (65)		
	Agree	9 (29)	2 (18.2)	7 (35)		
	Disagree	0 (0)	0 (0)	0 (0)		
	Strongly disagree	0 (0)	0 (0)	0 (0)		
	Missing	1 (3.2)	1 (9.1)	0 (0)		
**I do not have the technical skills to use digital technology with my patients**						.19
	Strongly agree	0 (0)	0 (0)	0 (0)		
	Agree	5 (16.1)	0 (0)	5 (25)		
	Disagree	20 (64.5)	8 (72.7)	12 (60)		
	Strongly disagree	5 (16.1)	2 (18.2)	3 (15)		
	Missing	1 (3.2)	1 (9.1)	0 (0)		
**Digitally produced devices always fit better**						.55
	Strongly agree	2 (6.5)	0 (0)	2 (10)		
	Agree	5 (16.1)	1 (9.1)	4 (20)		
	Disagree	22 (71)	9 (81.8)	13 (65)		
	Strongly disagree	0 (0)	0 (0)	0 (0)		
	Missing	2 (6.5)	1 (9.1)	1 (5)		
**3D printed devices enable high cost-effectiveness**						.39
	Strongly agree	2 (6.5)	0 (0)	2 (10)		
	Agree	15 (48.4)	4 (36.4)	11 (55)		
	Disagree	11 (35.5)	6 (54.6)	5 (25)		
	Strongly disagree	1 (3.2)	0 (0)	1 (5)		
	Missing	2 (6.5)	1 (9.1)	1 (5)		
**The future of prosthesis/orthosis industry and practice is digital**						.04
	Strongly agree	12 (38.7)	2 (18.2)	10 (50)		
	Agree	16 (51.6)	9 (81.8)	7 (35)		
	Disagree	3 (9.7)	0 (0)	3 (15)		
	Strongly disagree	0 (0)	0 (0)	0 (0)		

Singaporean P&Os who use technology agreed significantly less strongly (*P*=.04) than non-Singaporean P&Os that the future of prosthetics and orthotics is digital. Interviewees from Singapore suggested their current experience with technology has been both positive and negative, limiting their expectations for the future. They felt a need to use digital technology “for appropriate cases” or “when they improve efficiencies such as casting for a large transfemoral socket or making a scoliosis brace.” One interviewee stated that using digital software to “modify such large devices was more efficient and required less physical strength.”

**Figure 2:**
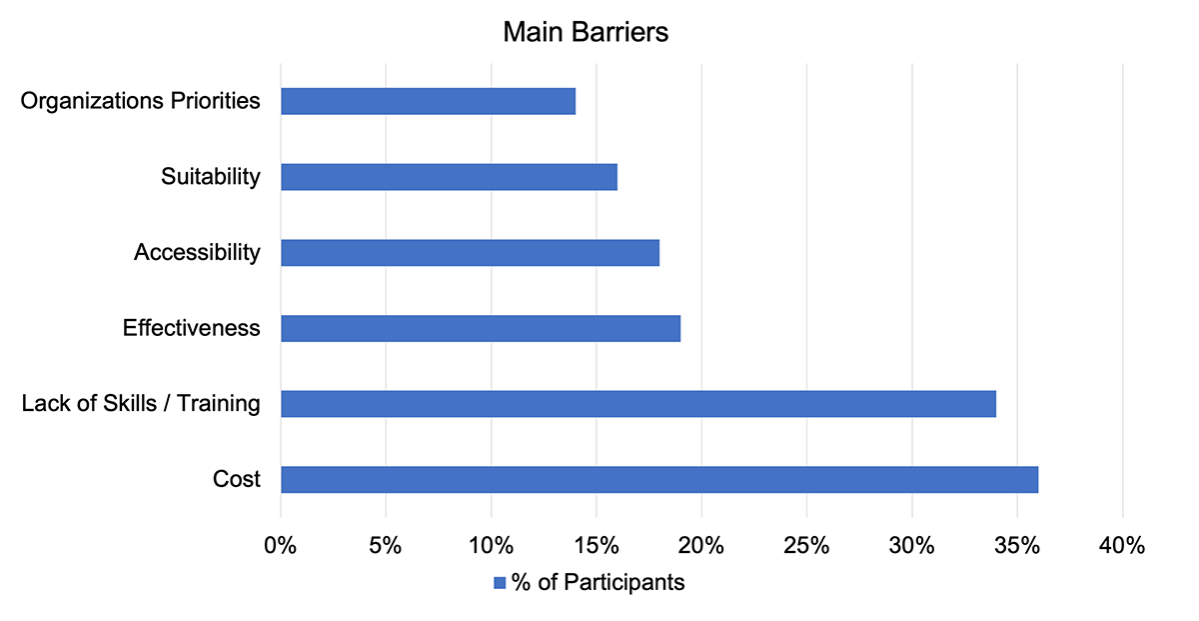
Barriers to greater integration of technology (prosthetist and orthotist who use technology, n=31).

The common barriers to greater integration of digital technology for the P&O-DT respondents as obtained using open-ended questions can be seen in Figure 2. The top barriers were cost (11/31, 35%) and the lack of skills and training (10/31, 32%). The third identified barrier was the effectiveness of technology (6/31, 19%). P&O-DT cited material strength, the need to outsource, and the constant software updates limiting the effectiveness of greater integration. These main barriers were similar to P&O-nonDT, highlighting an ongoing need for continual financial reinvestment and training even when digital services have been established.

### Nonuse of Technologies

Where nonuse of technology was common, stable internet was still a problem, particularly in developing countries such as Sri Lanka (2/3, 66%), Cambodia (2/5, 40%), and Myanmar (2/6, 33%), and many of the P&O respondents in these countries did not have computers (35/39, 89%). Other reasons mentioned for not using technology were cost (25/39, 64%) and the lack of awareness and skills (20/39, 51%).

### Virtual Care

The use of virtual assessments and virtual fittings were analyzed for agreement. A primary benefit of virtual services is to reach those who face obstacles in coming for their appointments. Out of 70 P&O respondents, 29 (41%) felt their patients had difficulties coming for their appointments. The main reasons mentioned were transportation (n=16, 19%), cost (n=11, 13%), and the lack of family members or caregivers to bring them to their appointment (n=9, 13%). P&O respondents found that virtual assessments would benefit the patient in these situations (n=59, 84%). Interestingly, 11 out of 13 (85%) LLA did not find access to care an issue and preferred to come to the clinic for their follow-ups even during the pandemic.

Out of 70 P&O respondents, 51 (73%) would use virtual assessments if it was made available. Most respondents agreed or strongly agreed that virtual assessments would be suitable in rural areas (n=47, 67%) but just over half suggest virtual fittings would improve patient outcomes (n=38, 54%). The potential benefits mentioned were to save clinical time and reduce the need to travel (n=32, 46%); this often reduces costs (n=17, 24%), and—of relevance during this current pandemic—10 (14%) suggested it would be safer for the patient and decrease the risk of infection.

Some confusion arose when P&O were asked about the format of the virtual assessments. Out of 7 selected interviewees, 5 (71%) revealed they had merely agreed to the statement without thinking how they might apply this service. Suggestions for the service included a “*triage-like*” service or checking “*simple things like whether all is well or not*” to “*assess the problem*” and “*determine whether a trip to the clinic was necessary*.” When asked if they felt patients would be willing to pay for this service, many “*did not think so*” unless “*it adds value*.” The LLA responses concurred with these statements. Only 6 of the 13 surveyed LLA are prepared to pay for this service, with 3 out of 3 (100%) of the LLA interviewees agreeing only if their needs were met.

The major potential challenges with virtual assessment mentioned by the P&O respondents were difficulties in assessing the limb for strength, ROM, palpation, and pain (26/70, 37%). Other problems were concerns of the skills the patient had to use for items such as computers (12/70, 17%), the high chance of miscommunication when giving advice (11/70, 16%), and internet connectivity (8/70, 12%).

Out of 70 P&O respondents, 27 (39%) were open to providing virtual fittings using a third person *fitter* with a further 15% considering it depending on the fitter’s skills and training. The main benefits cited were that it provides greater outreach and maintains the ability to overcome the common barriers like the need to travel to the clinic. When the P&Os were asked about patients doing the task of fitting themselves, safety concerns were mentioned during the interviews, despite LLA feeling confident in their ability (Table 6). There were mixed results for the level of confidence LLA have to adjust their own prosthesis with or without internet guidance. We found that those LLA who were less confident with internet guidance than by themselves, tended to be older than 45 years.

**Table 6:** The confidence of lower limb amputees adjusting their own prosthesis (n=13).

Confidence in adjusting the prosthesis	By self, n (%)	With internet guidance, n (%)
Extremely confident	4 (30.8)	4 (30.8)
Very confident	2 (15.4)	1 (7.7)
Somewhat confident	4 (30.8)	2 (15.4)
Not so confident	0 (0)	5 (38.5)
Not at all confident	3 (23)	1 (7.7)

## Discussion

### Principal Findings

To date, research has focused on the development of digital technologies or how new technology can be applied to the industry for a particular application. This survey reports the actual current use of digital technologies in the prosthetic and orthotic industry and suggests its suitability during pandemics such as COVID-19. Although infection prevention practices like social distancing, the wearing of masks, and regular washing of hands have been implemented, the use of digital technology for prosthetic and orthotic services remains challenging with many barriers to overcome. Current adoption levels of technology despite the pandemic suggest the potential benefits of safer care have not outweighed the limitations of the technology to provide sufficient value to both the patient and P&O. Furthermore, changing organizational behaviors in delivering digital health care require the right skills among health care professionals to leverage technology-driven solutions toward technology adoption.

### Use

Approximately half of the P&O respondents use some form of digital technology. The use of scanners, computers, and computer-augmented design and manufacturing are the most common ones. The use of scanners provides a mess-free and reduced physical contact environment, improving patient safety during the pandemic. There is still a need for the clinician to be present to conduct the scan; thus, only the physical touch component is improved.

The P&O respondents preferred the more cost-effective iPad with a structure scanner (Occipital) over high-end accurate scanners such as Vorum’s Spectra scanner or Artec EVA scanner. P&O interviewees stated that the wireless iPad was easier and lighter to maneuver to capture the limb shape but can be limiting when capturing the posterior view due to the screen’s position forcing an awkward posture of the person scanning. This finding is aligned with a study by Brunsman et al [48], where the positioning of the human body for surface scanning required an assortment of body postures to make all essential areas visible and the direction the patient faces can affect the quality of the scan. This repositioning may not reduce the prosthetist-patient contact as intended when trying to minimize cross-contamination, and it is lead author TB’s opinion, as a principal P&O with over 21 years of experience, that having a small handheld external camera connected via a cable or wirelessly to an external screen to view the captured image would be a simple solution to overcome these issues.

The use of low-cost cloud-based engineering modeling and analysis software programs such as Rhinoceros (Robert McNeel & Associates), Fusion360 (Autodesk), and Solidworks (Dassault Systèmes) was also common due to their affordability, usability, and applicability. Considering the P&O respondents stated that more training and skills are needed to increase adoption of technology, the use of point and click options in software [49] may remove the need for advanced CAD skills, making the technology more appealing and user-friendly [50]. This could lead to reduce unnecessary visits and contact with coworkers and patients, maintaining safe distancing and limiting possible virus spread.

Interviewees appreciated the improved efficiencies of digital scanning and software for the making of larger casts like transfemoral sockets or spinal braces. Stating that these types of casts can be modified using preloaded templates in the software in a shorter amount of time than physically removing or adding plaster via traditional methods. This process is more convenient and safer for the patient and faster for the P&O.

The use of 3D printing is often touted as the next big transition for the industry [51]. Our results suggest its use is relatively low. Three dimensional printing is similar to traditional production methods, where it is necessary to get throughput, part demand, and production planning right to minimize part manufacturing cost [52]. Three dimensional printing may change the way many products are developed and produced, and herald an era of “personal manufacturing” [53]. They also provide an efficient and safe manufacturing process; however, unless a facility is consistently 3D printing prostheses or orthoses, outsourcing is more economical.

### Barriers

The main barriers (cost, lack of skills or experience, and effectiveness of the technology) for adopting digital technology were found to be the same issues that prevented greater adoption in facilities already using some form of digital technology.

The initial cost outlay in purchasing scanners, computers, or 3D printers can cause apprehension over the return on investment. Interviewees reported that prosthetic- and orthotic-specific software requires special training, software updates, and yearly licensing, often based on the number of modules needed, adding to the cost and deterring more users from greater adoption. The use of 3D printing was found to be limited by the same factors identified in a systematic review of 3D printed sockets [51], including the quality of the part, choice of materials available, and the cost-effectiveness. Literature also points to associated costs of printing ignored when comparing to traditional methods, including the additional material costs for support structures, machine use rates, labor and print preparation, machine maintenance, and the error costs [54].

Even though the design and manufacturing of highly accurate prostheses and orthoses is possible with the help of digital technology, it was concerning to see that a majority of P&O who already used digital technology did not find the devices had a better fit. This result may be attributed to the need for ongoing training and practice to enhance the skills; most P&O were only using the technology for less than 5 years. Another reason could be the printing quality, which has increased over recent years but still requires the more expensive printers.

The use of scanning for AFOs was high but limitations in contactless scanning were voiced during the interviews as the P&O would often need to position the limb on a clear Perspex plastic scanning platform or the ground before scanning. The scanning of residual limbs for prosthetic sockets was easier, although—as previously discussed—positioning the scanner still remains troublesome to obtain a full 360° image with multiple positions needed to capture the entire shape [48].

Our survey suggests a low use of digital technology for transtibial socket design, with the LLA respondents complaining of poor design, fit, and ease of wearing their prostheses as major factors inhibiting their mobility. This is despite digital technology such as Finite Element Analysis, MRI, CT, and photogrammetry showing benefits to improve outcomes by predicting accurate interface pressures through better imaging of the muscles and tissues. It also allows further optimization in the design of comfortable high quality devices [55-58].

### Virtual Care

#### Patient

Out of 13 respondents, 11 (85%) of LLA did not find access to care an issue and preferred to come to the clinic for their follow-ups even during the pandemic. It should be noted all but 1 patient was from Singapore. Almost half of all P&O respondents outside Singapore found their patients had difficulties coming for their appointment. This is at odds with other countries where patients are more comfortable using telemedicine rather than risk infection with face-to-face consultations [59]. Our study did find support for virtual assessments from the P&O interviewees, who noted it was safer for patients and protected them from possible virus infections.

Questions remain about what types of tasks are suitable for virtual care particularly during the pandemic; all P&O interviewees suggested that triaging a patient or providing education to patients may be most suitable. The National Health Service program “Attend anywhere” suggests that virtual care is only useful if it results in improved efficiencies, significant time savings, reduced need to take time off work, no travel costs, and no technical issues [60]. Our study also showed the lack of IT skills and connection issues of the patients as concerns, highlighting the need for reliable infrastructure. Although virtual care would be an excellent solution for patients in remote areas and developing countries, this is also where infrastructure is likely to be poor. These results are aligned to Mihalj et al [9], who describes five factors that support telemedicine implementation. These include technology (broadband and connection) that must support both the health care provider and patient; secure platform; training to health care providers; patients’ need to be educated on privacy, safety, efficacy, and personal benefits; and cognitive and hearing impairments [9].

In a telehealth consumer study in the United States, the authors found that 66% of patients were willing to use telemedicine in 2019, but only 8% had used it previously. The authors suggest that the main reasons were the lack of familiarity with the new technology and a lack of trust in the clinician whom they have not met in person [61]. The emotional connection to the clinician is equally important in telehealth adoption. Knowing that the consultation focuses not only on the immediate health care needs but also the emotional support is critical to gaining loyalty from the patient [62].

This same issue of trust was highlighted in this study by both LLA and P&O respondents. In an industry that customizes devices, any change in the care model should reflect a strong need for such change. By merely moving consultations online, we may overcome some barriers found in this study, such as the travel burden, the lack of support to bring patients to their appointments, and reduced overall costs. However, there appears a need to develop a rapport between P&O and LLA before the use of virtual care and certain P&O tasks may be difficult to fulfill (see next section). A thoughtful application and design of digital technology is needed, considering all stakeholders involved.

#### P&Os

Literature suggests minimizing casting processes to prevent the virus spread [10]. The adoption of 3D scanning would be a viable method in achieving this. Concerns over how to conduct shape capturing, residual limb assessment, palpation, and gait analysis may limit the effectiveness and adoption of digital technology, unless it can be developed to overcome these challenges. The lack of touch and feel was found to be a major hurdle to adoption. Virtual assessment tools allow implementing triage at the point of need [63], but advice-only consultations may not prove valuable. LLA suggested they may be unwilling to pay for such services. Both LLA and P&O respondents are used to a consultation and physical contact combination. The information garnered through physical examinations, such as tissue consistency, identifying painful areas, or ROM, may prove challenging to overcome in a virtual setting.

In rural settings, our survey suggests the use of virtual care may be more suitable. This study found that P&Os would use virtual care where patients have to travel long distances for care or are too sick to come to a facility. However, in such rural locations, there may be other challenges such as internet connectivity and the IT skills of patients, limiting its applicability [9]. Our survey suggested the use of a third, local person to assist with data collection and fitting of devices, which might help to overcome some limitations. Attitudes toward the use of such persons were mixed. They would need sufficient competencies to ensure the appropriateness and quality of care. In the case of pandemic-related social distancing laws, the viability of such a third-person service would also be affected. Third person or support staff were used as a means to provide care in rural New South Wales, Australia, in combination with video calling for the provision of AFOs [39]. In this study, the authors trialed the assessment of the ROM over a video call with a third person performing the task. They found, when using the primary care giver as the third person, the measurements of the ROM were less accurate than the P&O. However, when a third person had a health care background the results were acceptable, suggesting a possible minimal educational background.

#### Hospital and Facility

The impetus for change and adoption of digital technology varies based on the funds and infrastructure available. Budgets may be too small to invest in digital technology and on training, government support may be low, and the use may be too infrequent to justify the investment. The purchase power to outsource may also present challenges, particularly if it is too low. Digital technology would be more widely adopted if it demonstrated enhanced patient care and outcomes, and lowered overheads of the facility, provided the infrastructure of the country can support the technology.

### Limitations

Our online survey was developed to obtain a broad understanding of digitalization in the P&O industry. Its length may have been the reason why 30 of the 113 respondents answered less than 10%. Furthermore, as this was an online survey, only respondents with internet connection were able to respond. This may have particularly affected the number of LLA responses; 12 of the 13 LLA respondents were from Singapore, contacted through the amputee support chat group, where internet connection is not a barrier. The P&O respondents may have been less affected, as they could have used the internet connections at work. Another reason for the low LLA response might be that they were contacted indirectly, via their P&O, or that multiple languages of the survey were not available.

The responses for Singapore are considered an accurate reflection of the P&O use of digitalization with over 65% of all P&O in Singapore participating. Although the other respondents came from a large number of countries, their numbers were limited. The study is, therefore, not representative of current practices outside of Singapore, even though the results are informative.

### Future Directions

Further investigation should focus on the exact nature of how virtual care during the pandemic can be conducted, particularly the lack of the element of touch in an assessment by the P&O. There is a clear need for the development of a digitalization framework to facilitate digital technology implementation in the industry. Understanding how, when, and why to use digital technology is vital for successful outcomes to both clinic and patient. Particular attention should be paid to delivery care models that overcome the shortfalls with current technology, including sensory feedback through palpation, low IT awareness, and poor connectivity, while maintaining safer care. The use of distributed care models (DCMs) is an alternative to switching all business to digital means. DCMs consist of a hybrid of care that includes central-based fabrication, satellite clinics, mobile clinics, and digital technology. Using a third person trained to digitalize the anatomy of a limb should be considered to enhance the outreach where prevailing laws allow.

### Conclusion

The use of digitalization during a pandemic such as COVID-19 can mitigate the concerns regarding ongoing patient care and safety for both patient and P&O. The use of scanning and virtual care provides avenues for the continuum of care for the patient. However, essential characteristics of P&O assessments such as palpation and sensory feedback have yet to be overcome. Providing the patient with the appropriate technology and answering what needs the technology is addressing is essential and may encourage adoption among the industry. Education and training should be provided to centers and individuals to enhance confidence levels and awareness of digital care benefits and risks during and beyond pandemic times. Ensuring the staff has a high technology readiness level is critical. The delivery care model should be evaluated to provide sufficient outreach and an optimal level of digital technology that provides adequate care and sufficient protection against the spread of COVID-19.

Technology advancements such as virtual platforms, digitalization methods, and improved connectivity will change the future of health care. Digital technology is transforming health care into a new normal and is being accelerated during the pandemic. This transformation is expected to continue in the years to come. The prosthetic and orthotic industry should keep an open mind and move toward creating the required infrastructure to support this digital transformation or risk being left behind.

## Abbreviations

AFO: ankle-foot orthosis
CAD: computer-aided design
CADCAM: computer-aided design and manufacturing
CT: computed tomography
DCM: distributed care model
IRB: Institutional Review Board
IT: information technology
LLA: lower limb amputees
MRI: magnetic resonance imaging
P&O: prosthetist and orthotist
P&O-DT: P&O who are currently using digital technology
P&O-nonDT: P&O who did not use digital technology
ROM: range of motion
